# Fermentation and Immobilization of Insect-Derived Deltamethrin-Degrading Strain, *Microbacterium* sp.

**DOI:** 10.3390/insects17010003

**Published:** 2025-12-19

**Authors:** Zhengyan Wang, Qiong Luo, Yifan Liu, Tianwei Ye, Yujia Zhang, Wei Xu

**Affiliations:** 1 School of Food and Strategic Reserves, Henan University of Technology, Zhengzhou 450001, China; zywangedu@163.com (Q.L.); fan3013492235@163.com (Y.L.); 13667419166@163.com (T.Y.); 2 College of Environmental and Life Sciences, Murdoch University, Perth, WA 6150, Australia; 18304019727@163.com (Y.Z.); w.xu@murdoch.edu.au (W.X.)

**Keywords:** *Microbacterium* sp., deltamethrin, biodegrade, immobilization, sodium alginate, environmental remediation

## Abstract

Extensive application of deltamethrin on agricultural products results in serious contamination of the environment. Its negative impact on environmental and public health necessitates the development of environmental remediation technologies. Detailed investigations of microbial degradation of deltamethrin may be useful for the development of bioremediation strategies. In this study, the deltamethrin removal capability of a bacterial strain, *Microbacterium* sp., previously isolated from the gut of *Tribolium castaneum* (Herbst) (Coleoptera: Tenebrionidae) was first investigated. When 3 mL of the bacterial solution (OD_600_ = 1) was inoculated into 97 mL of MS media containing 200 μg/mL deltamethrin, it could remove 45.7% deltamethrin after 64 h of incubation. This strain grew fastest in LB media with an inoculum volume of 3% in pH 7 at 175 rpm, 25 °C. To enhance its environmental tolerance, this strain was immobilized with sodium alginate. *Microbacterium* sp.-containing calcium alginate microspheres (CAMs) exhibited an enhanced deltamethrin removal capability compared to free bacteria, and CAMs generated by immobilization with 2% sodium alginate and 3% CaCl_2_ cross-linking for 4 h possessed the maximum deltamethrin removal capability. The ultrastructure of *Microbacterium* sp.-containing CAMs prepared under optimal conditions was a three-dimensional mesh structure with pores and dense features, and the bacteria grew well in the immobilized carrier. After being reused five times, the deltamethrin removal rate of immobilized *Microbacterium* sp. still reached over 50%. When *Microbacterium* sp. was inoculated into deltamethrin-contaminated water or soil for 48 h, the deltamethrin removal rate of immobilized bacteria was 1.4 times higher than that of free bacteria. These results suggest that *Microbacterium* sp.-containing CAMs possess an excellent deltamethrin removal capability and good reusability, showing great potential for the remediation of deltamethrin-contaminated environments.

## Simple Summary

In agricultural production, the extensive use of insecticides has led to the persistent presence of residues such as deltamethrin in the environment, posing threats to ecosystems and human health. Utilizing microbes for environmental bioremediation presents a feasible approach. Studies have found that insects with strong insecticide tolerance often harbor gut bacteria capable of degrading insecticides. These gut bacteria are becoming an excellent reservoir of insecticide-degrading bacteria with bioremediation potential. However, in practical applications, the activity of free bacterial cells is easily affected by environmental factors, limiting the stability of their remediation efficacy. This study focused on a *Microbacterium* sp. strain isolated from insects. By optimizing culture conditions and the sodium alginate immobilization process, the remediation efficiency of the immobilized strain in contaminated soil and water was investigated. The results demonstrated that the immobilized strain significantly outperformed free bacteria in deltamethrin degradation performance in both contaminated soil and water, while also exhibiting good stability and reusability. This research provides an effective method for the remediation of deltamethrin-contaminated environments.

## 1. Introduction

In modern agriculture, for the management of various pests and the production of high-yield crops, pesticides are extensively applied all over the world [1]. However, many studies have reported that pesticide residues are abundant in soils, sediments, and water bodies [2]. These hazardous compounds and their toxic metabolite residues significantly affect living organisms such as soil biota, fish, birds, mammals, plants, and human beings [3]. Their toxic residues interfere with organisms’ behavior, reproduction cycles, and metabolism mechanisms, which can permanently alter the interrelated ecosystems [4]. These toxic compounds are degraded into less toxic or even nontoxic substances using various methods such as chemical reactions, physical methods, photodegradation, and biodegradation. Compared to other techniques, biological methods, especially microbial remediation, are less expensive, environment-friendly, more effective, and easier to adapt to remove insecticide residues [5].

Insects constitute the largest group of organisms on earth, about 66% of all animal species, and gut bacteria can assist insect hosts to degrade insecticides [6,7]. It has been proven that insect pests with strong insecticide tolerance are an excellent reservoir of insecticide-degrading bacteria with bioremediation potential [8]. At present, insecticide-degrading bacteria have been successfully isolated from the gut of various insects. For example, several gut bacterial strains capable of degrading deltamethrin, pirimiphos-methyl, and malathion were isolated from stored product beetles [9,10]. However, the performance of free bacteria can be affected by abiotic and biotic factors in practical applications that can lead to reduced insecticide removal activity. Abiotic factors include temperature, moisture, pH, and organic matter content. Biotic factors include competition between indigenous and exogenous microbes for limited carbon sources, as well as antagonistic interactions and predation by protozoa and bacteriophages [11].

Microbial immobilization technologies could overcome these drawbacks by retaining or confining free microbial cells to a specific space, and could keep the microbes active in adverse conditions for a longer period [12]. The choice of material used for immobilizing microbes is crucial as it influences their viability and the effectiveness of xenobiotic removal. Three types of carriers are available: organics, which can be further divided into natural (e.g., chitin, agar, alginate, and carrageenan) and synthetic organics (e.g., polyvinyl alcohol, polypropylene ammonium, polyurethane, and acrylamide), and inorganics (e.g., activated carbon, clay, zeolite, anthracite, ceramics, and porous glass) [13]. Among them, sodium alginate is the most widely used carrier in water treatment, as it forms microspheres when mixed with microbes and calcium [14]. For example, the removal efficiency of benzene hexachloride in water by *Pseudomonas fluorescens* immobilized with sodium alginate and CaCl_2_ could reach up to 81.8%, and immobilized *P*. *fluorescens* could withstand the environmental stress more easily and for a longer time than free *P*. *fluorescens* [15].

Deltamethrin is one of the most frequently and widely used pyrethroids against stored product, household, crop, and forest pests. Deltamethrin residues are retained in many environmental matrices, such as agricultural products, soils, atmosphere, surfaces, and groundwater, and have a relatively long half-life due to their low solubility in water. Deltamethrin shows neurotoxicity, reproductive toxicity, and immunotoxicity, and acts as an endocrine disruptor against non-target organisms [16]. Its negative impact on environmental and public health necessitates the development of environmental remediation technologies. Although several deltamethrin-degrading microbes from different genera, such as *Bacillus* [10,17], *Brevibacillus* [18], and *Stenotrophomonas* [16], have been isolated and identified, it has been reported that some of them pose potential biosafety risks and require careful consideration in environmental remediation practice [19]. Therefore, new bacterial strains need to be screened to enlarge the gene pool for the construction of pesticide-degrading, engineered bacteria and provide more microbial candidates for environmental bioremediation technologies.

In our previous study, a novel bacterial strain, *Microbacterium* sp., isolated from the gut of adult *Tribolium castaneum* (Herbst) (Coleoptera: Tenebrionidae), grew well in MS media containing deltamethrin as the sole carbon source. Thus, its potential for deltamethrin degradation was further evaluated in this study. Here, we first investigated its deltamethrin removal capability. Due to its prominent performance in deltamethrin biodegradation, we further figured out its optimal culture conditions. Next, the conditions for immobilizing *Microbacterium* sp. with sodium alginate were optimized, aiming to improve its application efficacy under natural conditions. Finally, the deltamethrin removal capability of immobilized bacteria in deltamethrin-contaminated soil and water was investigated under indoor simulated conditions.

## 2. Materials and Methods

### 2.1. Culture Media and the Bacterial Strain

Luria–Bertani (LB) media were composed of 10.0 g/L peptone (Sangon Biotech, Shanghai, China), 5.0 g/L yeast extract (Sangon Biotech, Shanghai, China), and 10.0 g/L NaCl (Macklin, Shanghai, China), and adjusted to a certain pH with 1 mol/L NaOH (Macklin, Shanghai, China) or HCl (Macklin, Shanghai, China) for optimizing its culture conditions. Minimal salt (MS) media were composed of 1.0 g/L NaCl, 1.0 g/L (NH_4_)_2_SO_4_ (Macklin, Shanghai, China), 1.5 g/L K_2_HPO_4_ (Macklin, Shanghai, China), 0.5 g/L KH_2_PO_4_ (Macklin, Shanghai, China), 0.1 g/L MgSO_4_·7H_2_O (Macklin, Shanghai, China), and 0.01 g/L FeSO_4_·7H_2_O (Macklin, Shanghai, China).

The bacterial strain used in this study was isolated from the gut of adult *T*. *castaneum* and identified as *Microbacterium* sp. by 16S rDNA sequence similarity analysis [10], with accession number OM992229.1 in the NCBI database. The bacterial strain was cultured with LB media at 150 rpm, 37 °C for 24 h, and then centrifuged at 5000 rpm, 30 °C for 10 min (Centrisart D-16C, Gottingen, Germany). The precipitate was washed twice with phosphate buffer solutions (pH 7.2) and then resuspended with deionized water to OD_600_ = 1, which was used as the stock bacterial solution for the following tests.

### 2.2. Evaluation of the Deltamethrin Removal Rate

The stock bacterial solution was mixed with MS media containing 200 μg/mL deltamethrin (Tmrm, Beijing, China) at the volume ratio of 3: 97 (i.e., inoculum volume 3%) and incubated at 175 rpm, 25 °C for 64 h. The MS media containing deltamethrin only served as a control. An aliquot of 2 mL of the sample was collected after 0, 8, 16, 24, 32, 40, 48, 56, and 64 h of incubation. The sample was centrifuged at 5000 rpm, 4 °C for 10 min (Eppendorf 5430R, Hamburg, Germany), and the supernatant was collected and subjected to liquid–liquid extraction using an equal volume of *n*-hexane (HPLC grade, Aladdin, Shanghai, China). The organic phase was concentrated with a nitrogen blow-dryer (DN-12A, Bilon Instrument, Shanghai, China). The concentrate was diluted with *n*-hexane to 1 mL for GC-MS analysis. The analysis was performed with a GC-2010 Plus gas chromatograph (Shimadzu, Kyoto, Japan) equipped with a 30 m × 250 μm i.d. DB-5 MS UI capillary column (Agilent, Beijing, China) and a GC-MS-QP2010 Ultra inert mass spectrometry detector with electron impact ionization (Shimadzu, Kyoto, Japan) according to the method of Wang et al. 9. The external standard method was used to calculate the deltamethrin content in each sample, and the deltamethrin removal rate was calculated by the following formula:

*ρ* = (1 − A1/A0) × 100%

where A1 is the content of deltamethrin in MS media incubated with bacteria, and A0 is the content of deltamethrin in the control. Each treatment had five replicates.

### 2.3. Optimization of Bacterial Culture Conditions

Four independent variables, namely the inoculum volume of the stock bacterial solution, temperature, rotation speed of the shaker, and initial pH of the culture media, were adopted to optimize the conditions for culturing *Microbacterium* sp. with LB media. The experimental design involved changing one variable at a time while keeping others at a fixed level, and the fixed variables were an inoculum volume of 3%, 30 °C, a rotation speed of 150 rpm, and an initial pH of 7, which facilitated the rapid growth of most microbes [20,21]. The levels of each variable were as follows: inoculum volume, 1%, 2%, 3%, 4%, and 5%; temperature, 20, 25, 30, 35, and 40 °C; rotation speed, 100, 125, 150, 175, and 200 rpm; and initial pH, 5, 6, 7, 8, and 9 [22]. After optimal level ranges of each variable were preliminarily determined, the orthogonal L_9_(3)^4^ test on three levels of these four variables (Table 1) was further used to optimize culture conditions of *Microbacterium* sp. The OD_600_ value of the bacterial solution was measured with an ultraviolet spectrophotometer (Eppendorf D30, Hamburg, Germany) after 32 h of cultivation, by which time the growth of the bacteria had not entered the stationary phase yet. Each treatment had five replicates.

### 2.4. Bacterial Immobilization

Bacteria were immobilized in calcium alginate microspheres (CAMs) using the method described by Khalid et al. [23] with minor modifications. An aliquot of 50 mL of the stock bacterial solution was centrifuged at 5000 rpm, 30 °C for 10 min. The precipitate was resuspended in 4 mL of deionized water, then thoroughly mixed with 100 mL of an aqueous sodium alginate (Sangon Biotechnology, Shanghai, China) solution, and then the *Microbacterium* sp.-containing sodium alginate solution was dropped in aqueous CaCl_2_ solutions for different periods (i.e., cross-linking time) for cross-linking between calcium alginate to generate *Microbacterium* sp.-containing CAMs. The microspheres were then dipped in 5% KH_2_PO_4_ for hardening, and then transferred to and stirred magnetically in deionized water three times to remove extra CaCl_2_ and phosphate. The purified microspheres were dried on a sterile clean bench and then stored at 4 °C for the following tests.

Three independent variables, namely the concentration of sodium alginate and CaCl_2_ and cross-linking time of calcium alginate, were adopted to optimize the conditions for immobilizing *Microbacterium* sp. The experimental design involved changing one independent variable at a time while keeping others at a fixed level, and the fixed variables were 3% sodium alginate, 3% CaCl_2,_ and cross-linking for 3 h, which were claimed as the optimal conditions for the formation of sodium alginate–chitosan gel microspheres [24]. The levels of each variable were as follows: sodium alginate concentration, 1%, 2%, 3%, 4%, and 5%; CaCl_2_ concentration, 1%, 2%, 3%, 4%, and 5%; and cross-linking time, 1, 2, 3, 4, and 5 h [25]. After optimal level ranges of each variable were preliminarily determined, the orthogonal L_9_(3^3^) test on three levels of these three variables (Table 2) was further used to optimize the immobilization conditions.

To evaluate the effect of the immobilization conditions on the deltamethrin removal capability of immobilized *Microbacterium* sp., 30 CAMs produced under each immobilization condition or the same biomass of free bacteria were inoculated into 100 mL of MS media containing 200 μg/mL deltamethrin, and the residual deltamethrin in MS media was quantified after 48 h incubation at 175 rpm, 25 °C. The MS media containing deltamethrin only served as a control. After incubation, the residual deltamethrin in the culture media was quantified, and its removal rate was calculated according to the above-mentioned method. Each treatment had five replicates.

As CAMs generated by immobilization with 2% sodium alginate and 3% CaCl_2_ cross-linking for 4 h exhibited the maximum deltamethrin removal capability, they were used in the following tests.

### 2.5. Characterization of Immobilized Microbacterium sp.

To visualize the immobilization state of *Microbacterium* sp. in CAMs, bacteria-containing CAMs were washed three times with phosphate buffer solution (pH 7.2). CAMs were cut using a sterilized knife, fixed with 2.5% glutaraldehyde at 4 °C for 2 h and then dehydrated by ethanol with a concentration gradient of 30%, 50%, 70%, 80%, 90%, and 95% for 15 min, followed by dehydration with acetone and drying with supercritical carbon dioxide. Then, samples were mounted on specimen tubs and successively painted with silver and gold, and then examined using a scanning electron microscope (S8100, Hitachi, Tokyo, Japan) [26].

To evaluate the optimal application dosage of the CAMs to remove deltamethrin, 30, 40, 50, 60, and 70 *Microbacterium* sp.-containing CAMs were inoculated in 100 mL of MS media supplemented with 200 μg/mL deltamethrin at 175 rpm, 25 °C for 48 h. To assess the reusability of the CAMs to remove deltamethrin, 40 *Microbacterium* sp.-containing CAMs were inoculated in 100 mL of MS media supplemented with 200 μg/mL deltamethrin at 175 rpm, 25 °C for 48 h, then the CAMs were filtered out and washed with deionized water, and then inoculated in new deltamethrin-containing MS media at 175 rpm, 25 °C for 48 h. This process was repeated four times. The MS media containing deltamethrin only served as a control. After each incubation, the residual deltamethrin in the culture media was quantified, and its removal rate was calculated according to the abovementioned method. Each treatment had five replicates.

### 2.6. Application of the CAMs Under Mimic Situations

To assess the deltamethrin removal capability of *Microbacterium* sp.-containing CAMs in deltamethrin-contaminated water, 800 *Microbacterium* sp.-containing CAMs or the same biomass of free bacteria were added to 1 L of distilled water supplemented with 100 μg/mL deltamethrin. Distilled water supplemented with deltamethrin only served as a control. All samples were incubated at 175 rpm, 25 °C, and 2 mL of samples were collected after 0, 6, 12, 18, 24, 30, 36, 42, and 48 h of incubation [26]. The residual deltamethrin in samples was quantified, and its removal rate was calculated according to the abovementioned method. Each treatment had five replicates.

To assess the deltamethrin removal capability of *Microbacterium* sp.-containing CAMs in deltamethrin-contaminated soil, 800 *Microbacterium* sp.-containing CAMs or the same biomass of free bacteria were mixed with 2 kg of non-sterile soil, which was sampled from the campus of Henan University of Technology and preliminarily mixed with deltamethrin at a dosage of 100 mg/kg. Non-sterile soil containing deltamethrin only served as a control. All samples were incubated at 25 °C, and 5 g of soil was sampled after 0, 6, 12, 18, 24, 30, 36, 42, and 48 h of incubation. The soil sample was extracted twice with 10 mL of *n*-hexane and centrifuged at 3000 rpm, 4 °C for 3 min, and approximately 4 mL of supernatant was obtained. The supernatant was blow-dried with the nitrogen blow-dryer and then reconstituted with 2 mL of *n*-hexane [27]. The residual deltamethrin in soil samples was quantified, and its removal rate was calculated according to the abovementioned method. Each treatment had five replicates.

### 2.7. Statistical Analysis

The results from the orthogonal test were analyzed with extreme difference analysis. The statistical significance of differences between the means from different cultures and immobilization conditions, application dosages, repeated application times of the CAMs, and incubation times of the CAMs in deltamethrin-contaminated water and soil were evaluated with a one-way analysis of variance (ANOVA) followed by Tukey’s honestly significant difference (HSD) test. The statistical difference between the means of the treatments of free and immobilized bacteria was analyzed with Student’s *t*-test. For all analyses, if not specially mentioned, the statistical significance criterion was set at *p* < 0.05. All statistics were performed with SPSS Statistics version 26.0, and GraphPad Prism 8.0.2 was used to plot the experimental data.

## 3. Results

### 3.1. Deltamethrin Removal Capability of Microbacterium sp.

**Figure 1:**
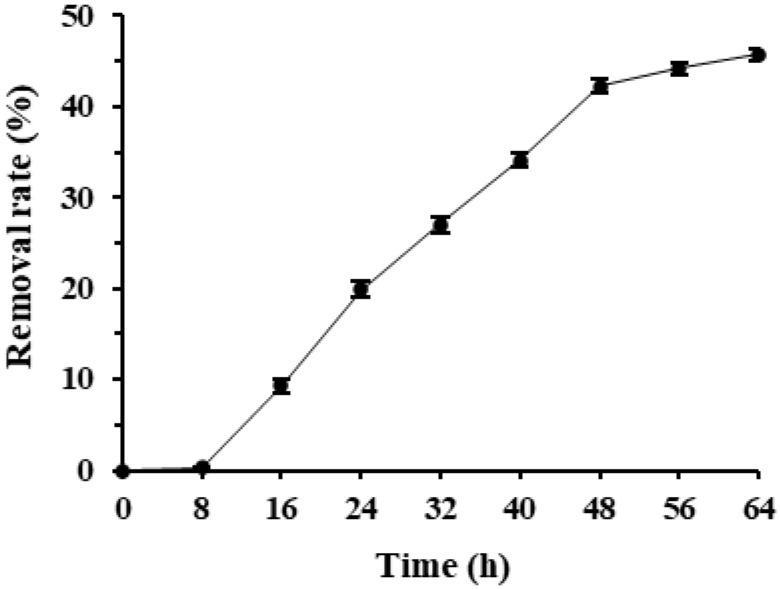
Mean ± SE deltamethrin removal rate during 64 h incubation of *Microbacterium* sp. in MS media with the bacterial inoculum volume of 3% at 175 rpm, 25 °C (*n* = 5).

Deltamethrin removal rates in *Microbacterium* sp.-containing MS media increased quickly over time and reached a high level after 48 h of incubation. *Microbacterium* sp. entered the rapid deltamethrin degradation period after 8 h of incubation and removed 45.7% deltamethrin after 64 h of incubation (Figure 1). As deltamethrin was the only carbon source in the culture media, this suggests that *Microbacterium* sp. has great potential in degrading and utilizing deltamethrin as the sole carbon and energy source for growth. As the deltamethrin removal rate of *Microbacterium* sp. did not reach a plateau within 32 h of incubation and slowed down after 48 h of incubation, 32 and 48 h were selected as the incubation duration to optimize its culture conditions and to evaluate its deltamethrin removal capability, respectively, in the following tests.

### 3.2. Optimal Culture Conditions of Microbacterium sp.

**Figure 2:**
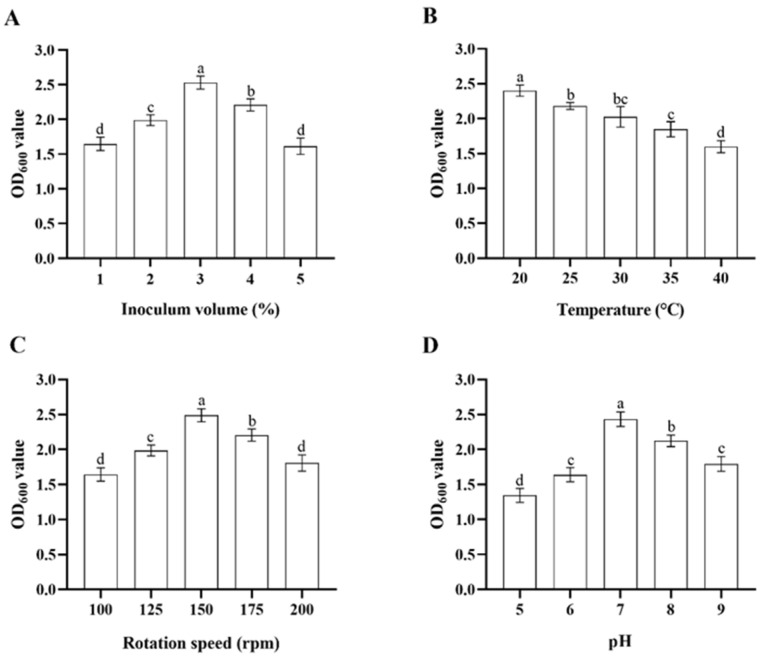
Mean ± SE OD_600_ value of LB media after 32 h incubation with *Microbacterium* sp. with different bacterial inoculum volume (**A**), temperature (**B**), rotation speed of the shaker (**C**), and initial pH of the culture media (**D**) (*n* = 5). The same letters indicate that there is no significant difference among means from different treatments (one-way ANOVA and Tukey’s HSD, *p* > 0.05).

In single-variable experiments, the inoculum volume of the stock bacterial solution, temperature, rotation speed of the shaker, and initial pH of the culture media significantly influenced the growth of *Microbacterium* sp. in LB media. When incubated at a pH of 7 at 150 rpm, 30 °C, *Microbacterium* sp. grew fastest with an inoculum volume of 3%, followed by inoculum volumes of 4%, 2%, 1%, and 5% (ANOVA: *F*_4,20_ = 82.4, *p* < 0.001) (Figure 2A). When incubated with the inoculum volume of 3% in pH 7 at 150 rpm, *Microbacterium* sp. grew fastest at 20 °C, followed by 25, 30, 35, and 40 °C (ANOVA: *F*_4,20_ = 47.1, *p* < 0.001) (Figure 2B). When incubated with the inoculum volume of 3% in pH 7 at 30 °C, *Microbacterium* sp. grew fastest with a rotation speed of 150 rpm, followed by rotation speeds of 175, 125, 200, and 100 rpm (ANOVA: *F*_4,20_ = 61.4, *p* < 0.001) (Figure 2C). When incubated with the inoculum volume of 3% at 150 rpm, 30 °C for 32 h, *Microbacterium* sp. grew fastest in pH 7, followed by pH 8, 9, 6, and 5 (ANOVA: *F*_4,20_ = 92.0, *p* < 0.001) (Figure 2D). The top three levels of each variable that were suitable for the growth of *Microbacterium* sp. were selected for further orthogonal testing (Table 1).

Extreme difference analysis of results from the orthogonal test indicated that the influence of the four culture condition variables on the growth of *Microbacterium* sp., based on the *R* values from largest to smallest, was as follows: inoculum volume > rotation speed > temperature > pH (Table 3). The inoculum volume was the most important determinant for the growth of *Microbacterium* sp., and the bacterium grew fastest with an inoculum volume of 3% in pH 7 at 175 rpm, 25 °C.

### 3.3. Optimal Immobilization Conditions for Microbacterium sp.

**Figure 3:**
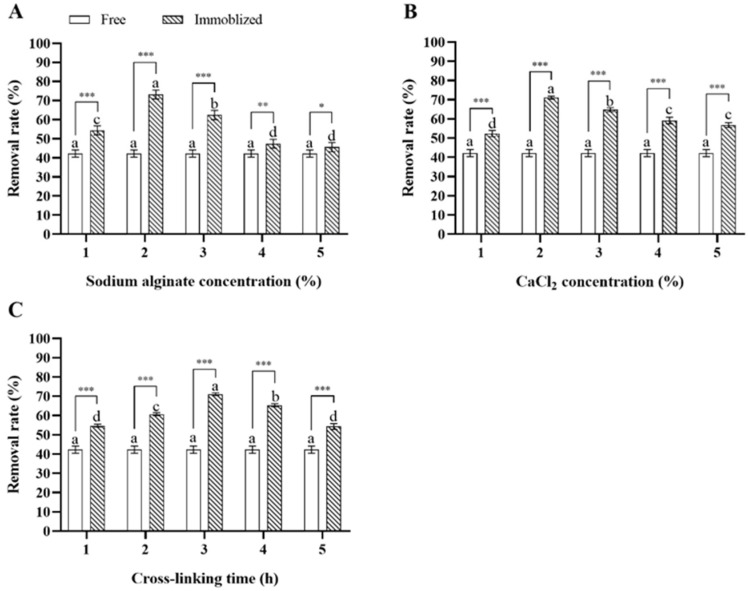
Mean ± SE deltamethrin removal rate in MS media after 48 h incubation with *Microbacterium* sp. immobilized with different concentrations of sodium alginate (**A**), CaCl_2_ (**B**), and cross-linking times of calcium alginate (**C**) (*n* = 5). The same letters indicate that there is no significant difference among means from the same bacterial treatment (one-way ANOVA and Tukey’s HSD, *p* > 0.05). * means that there is a significant difference between means in the same cluster (* means *p* < 0.05, ** means *p* < 0.01, and *** means *p* < 0.001) (Student’s *t*-test).

In single-variable experiments, immobilized *Microbacterium* sp. generally showed a higher deltamethrin removal capability compared to free *Microbacterium* sp., and the concentrations of sodium alginate and CaCl_2_ and the cross-linking time of calcium alginate significantly influenced the deltamethrin removal capability of *Microbacterium* sp.-containing CAMs. As for immobilization with 3% CaCl_2_ for 3 h, the deltamethrin removal rate in MS media inoculated with *Microbacterium* sp.-containing CAMs immobilized with 2% sodium alginate was the highest, followed by those with 3%, 1%, 4%, and 5% sodium alginate (ANOVA: *F*_4,20_ = 114.2, *p* < 0.001) (Figure 3A). As for immobilization with 3% sodium alginate for 3 h, the deltamethrin removal rate in MS media inoculated with *Microbacterium* sp.-containing CAMs immobilized with 2% CaCl_2_ was the highest, followed by those with 3%, 4%, 5%, and 1% CaCl_2_ (ANOVA: *F*_4,20_ = 149.2, *p* < 0.001) (Figure 3B). As for immobilization with 3% sodium alginate and 3% CaCl_2_, the deltamethrin removal rate in MS media inoculated with *Microbacterium* sp.-containing CAMs immobilized for 3 h was the highest, followed by those with the cross-linking times of 4, 2, 1, and 5 h (ANOVA: *F*_4,20_ = 278.6, *p* < 0.001) (Figure 3C). The top three levels of each variable that were most suitable for *Microbacterium* sp. immobilization were selected for further orthogonal testing (Table 2).

Extreme difference analysis of results from the orthogonal test indicated that the influence of three immobilization condition variables on the deltamethrin removal capability of *Microbacterium* sp.-containing CAMs, based on the *R* values from largest to smallest, was as follows: concentration of sodium alginate > concentration of CaCl_2_ > cross-linking time. The concentration of sodium alginate was the most important determinant of the deltamethrin removal capability of *Microbacterium* sp.-containing CAMs, and the CAMs with the maximum deltamethrin removal capability were generated by immobilization with 2% sodium alginate and 3% CaCl_2_ for 4 h (Table 4).

**Figure 4:**
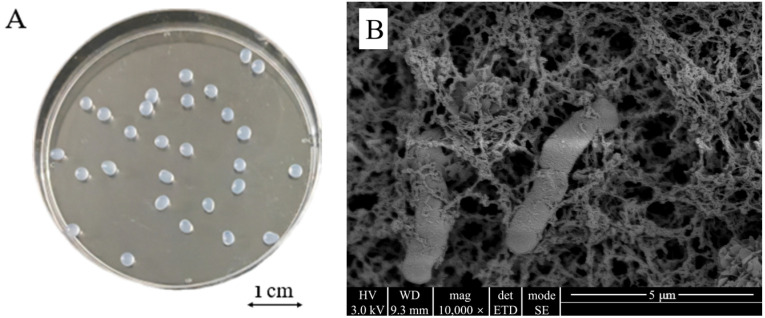
Morphology (**A**) and microscopic structure (**B**) of *Microbacterium* sp. immobilized in calcium alginate microspheres.

The outer surface of the CAMs prepared under optimal immobilization conditions was spherical, smooth, and had a diameter of approximately 2 mm and no tail (Figure 4A). The internal structure was a three-dimensional mesh structure with pores and dense features (Figure 4B). Intact cells of *Microbacterium* sp. were attached to the skeleton and pores inside the carrier and showed no apparent morphological changes upon immobilization, indicating that the bacteria grew well in the immobilized carrier. Such a structure increases the specific surface area of the carrier, which reduces the diffusion resistance and facilitates the transport of oxygen and nutrients.

**Figure 5:**
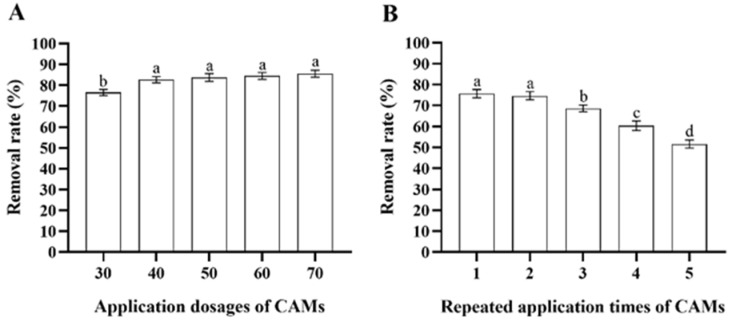
Mean ± SE deltamethrin removal rate in MS media after 48 h incubation with *Microbacterium* sp. immobilized in calcium alginate microspheres (CAMs) applied at different application dosages (**A**) and repeated application times (**B**) (*n* = 5). The same letters indicate that there is no significant difference among means from different treatments (one-way ANOVA and Tukey’s HSD, *p* > 0.05).

After incubation with 100 mL of MS media containing 200 μg/mL deltamethrin for 48 h, the deltamethrin removal rate in MS media inoculated with *Microbacterium* sp.-containing CAMs increased as more CAMs were added (ANOVA: *F*_4,20_ = 23.7, *p* < 0.001). However, there was no significant difference among the deltamethrin removal rates in MS media inoculated with 40, 50, 60, and 70 CAMs (Figure 5A). Therefore, considering cost-efficiency, 40 CAMs were recommended to degrade equivalent deltamethrin residue. Although the deltamethrin removal rates in MS media inoculated with *Microbacterium* sp.-containing CAMs decreased with increasing repeated application times of the CAMs (ANOVA: *F*_4,20_ = 137.0, *p* < 0.001), it remained above 50% when the CAMs were used for the fifth time (Figure 5B). This suggests that the immobilization of *Microbacterium* sp. with CAMs facilitates the recycling and reuse of *Microbacterium* sp.

### 3.4. Deltamethrin Removal Capability of Microbacterium sp. in Water and Soil

**Figure 6:**
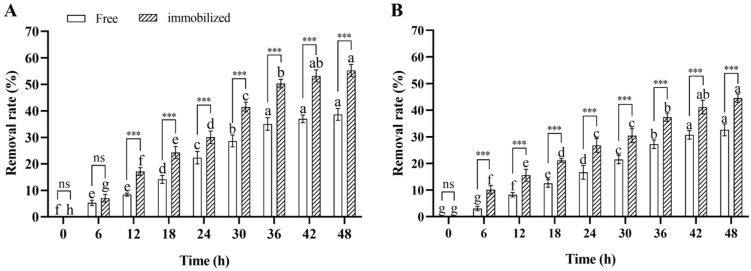
Mean ± SE deltamethrin removal rate in water (**A**) and soil (**B**) after 48 h incubation with free or immobilized *Microbacterium* sp. (*n* = 5). The same letters indicate that there is no significant difference among means from the same bacterial treatment (one-way ANOVA and Tukey’s HSD, *p* > 0.05). *** means that there is a significant difference between means in the same cluster (*p* < 0.001), while ns means that there is no significant difference (*p* > 0.05) (Student’s *t*-test).

In the water remediation experiment, the deltamethrin removal rate in contaminated water inoculated with immobilized and free *Microbacterium* sp. significantly increased over time during 48 h incubation (ANOVA: *F*_8,36_ = 605.2, *p* < 0.001 for immobilized; *F*_8,36_ = 358.1, *p* < 0.001 for free), and the deltamethrin removal rates in water inoculated with immobilized and free *Microbacterium* sp. after 48 h of incubation were 55.3% and 38.7%, respectively. This suggests that the deltamethrin removal capability of *Microbacterium* sp. was stable in contaminated water. Furthermore, the deltamethrin removal rate in water inoculated with immobilized *Microbacterium* sp. was higher than in water inoculated with free *Microbacterium* sp. after 12–48 h of incubation (Student’s *t*-test: *t*_8_ = 12.3, *p* < 0.001 for 12 h; *t*_8_ = 8.4, *p* < 0.001 for 18 h; *t*_8_ = 5.1, *p* < 0.001 for 24 h; *t*_8_ = 9.9, *p* < 0.001 for 30 h; *t*_8_ = 11.9, *p* < 0.001 for 36 h; *t*_8_ = 13.5, *p* < 0.001 for 42 h; *t*_8_ = 11.7, *p* < 0.001 for 48 h) (Figure 6A). This suggests that the deltamethrin removal capability of immobilized *Microbacterium* sp. was higher than that of free *Microbacterium* sp. in contaminated water.

In the soil remediation experiment, the deltamethrin removal rate in contaminated soil inoculated with immobilized or free *Microbacterium* sp. significantly increased over time during 48 h incubation (ANOVA: *F*_8,36_ = 307.5, *p* < 0.001 for immobilized; *F*_8,36_ = 282.7, *p* < 0.001 for free), and the deltamethrin removal rates in soil inoculated with immobilized and free *Microbacterium* sp. after 48 h of incubation were 44.5% and 32.6%, respectively. This suggests that the deltamethrin removal capability of *Microbacterium* sp. was stable in contaminated soil. Furthermore, the deltamethrin removal rate in soil inoculated with immobilized *Microbacterium* sp. was higher than in soil inoculated with free *Microbacterium* sp. after 6–48 h of incubation (Student’s *t*-test: *t*_8_ = 8.8, *p* < 0.001 for 6 h; *t*_8_ = 6.9, *p* < 0.001 for 12 h; *t*_8_ = 11.7, *p* < 0.001 for 18 h; *t*_8_ = 6.3, *p* < 0.01 for 24 h; *t*_8_ = 6.7, *p* < 0.001 for 30 h; *t*_8_ = 10.1, *p* < 0.001 for 36 h; *t*_8_ = 7.8, *p* < 0.001 for 42 h; *t*_8_ = 9.7, *p* < 0.001 for 48 h) (Figure 6B). This suggests that the deltamethrin removal capability of immobilized *Microbacterium* sp. was higher than that of free *Microbacterium* sp. in contaminated soil.

## 4. Discussion

In our previous study, it was found that *Microbacterium* sp. grew well in MS media containing deltamethrin as the sole carbon source. In this study, it was also found that *Microbacterium* sp.-containing CAMs still possessed the potential to degrade deltamethrin after being reused five times in MS media. This suggests that this bacterial strain degraded deltamethrin completely and assimilated it for its own biomass production. The degradation of pyrethroid pesticides by microbes generally follows an enzymatic mechanism common to many pesticide classes, in which secreted enzymes cleave carboxylic acid ester bonds to yield smaller carboxylic acids and alcohols. These intermediates are further oxidized or dehydrogenated, yielding compounds with lower toxicity or non-toxic substances [28,29]. Thus, it is speculated that there is little chance for deltamethrin to be degraded into more toxic substances by *Microbacterium* sp., which needs to be verified by further chemical analysis of the degradation products.

There are only a few reports of deltamethrin-degrading strains, because this insecticide is resistant to biodegradation due to the antimicrobial activities of 3-phenoxybenzaldehyde, a major hydrolysis product from the degradation of deltamethrin [30]. Although several deltamethrin-degrading microbes of different genera have been reported, *Microbacterium* appeared to be a new genus that was found to be highly effective in degrading deltamethrin. Wu et al. [16] reported that *Stenotrophomonas maltophilia* could degrade 63.3% of 100 μg/mL deltamethrin after 10 d of incubation, while *Microbacterium* sp. could degrade 45.7% of 200 μg/mL deltamethrin after 64 h of incubation. *Microbacterium* sp. can degrade deltamethrin in MS media after 8 h of incubation without being supplemented with other carbon sources, indicating that this strain adapted quickly to the environment. Furthermore, this strain grew well in LB media over a wide range of temperatures (20–40 °C) and pHs (5–9), particularly at low temperatures and pHs. This is an important feature for a microbe that is to be employed for the bioremediation of variable environments because the biodegradation of xenobiotics in the environment rarely occurs at high or low temperatures and pHs [31,32]. These prominent characteristics make *Microbacterium* sp. a promising candidate for the remediation of deltamethrin-contaminated environments.

Large-scale fermentative production is the first step for the application of functional microbes. As most bacteria grow well in LB media, it was used as the culture medium in this study. It has been reported that the growth of the bacteria is highly sensitive to variations in the initial inoculum size of the stock bacteria, initial pH of the culture media, rotation speed of the shaker, and temperature [21]. In this study, the influence of the four culture condition variables on *Microbacterium* sp. growth from largest to smallest was as follows: inoculum volume > rotation speed > temperature > pH. pH was the factor with the least influence. Considering that the intracellular pH of bacteria is close to neutrality and remains almost constant to preserve metabolic capacity and cellular integrity, our results suggest that *Microbacterium* sp. has a strong capability to sense and adapt to extracellular pH [33]. The high cost of LB media made it unsuitable for large-scale industrial fermentation. Fermentative production costs could be reduced by replacing expensive components with cheaper sources and/or by increasing productivity [34]. In the future, the medium formulations should be evolved by screening more economical or effective carbon and nitrogen sources.

In practical applications, the proliferation of functional microbes is affected by many abiotic and biotic factors that can lead to reduced insecticide removal activity [11]. Immobilization provides higher cell density and greater stability for bacteria and is a feasible approach to improve survival and retention of the functional microbes in contaminated sites. Additionally, the immobilization of microbes on a support matrix can reduce the cost of regeneration and recirculation and minimize bacterial loss [35]. Sodium alginate polymers have unique advantages for water treatment due to their ability to form gels and their biocompatibility [36]. They contain many hydroxyl groups and have good hydrophilicity, so microbes can attach to the molecular chain of the polymer. In addition, the three-dimensional space network structure also provides a good channel for the transfer and exchange of matter [37]. This study found that the internal structure of the CAMs was a three-dimensional mesh structure, indicating a high specific surface area, which provides a better microenvironment for the growth of bacteria and mass transfer of contaminants [38]. Usually, the inoculum size is an important factor determining the efficient biodegradation of the applied insecticides [30]. When the application dosage of the CAMs reached a certain level, its deltamethrin removal efficiency did not increase any more, verifying high mass transfer efficiency between the CAMs and the aqueous deltamethrin-contaminated system.

Some processing factors, such as the concentrations of sodium alginate and CaCl_2_ and the cross-linking time, influence the intraparticle structure and physical characteristics of the CAMs. This study found that the deltamethrin removal rate of the CAMs was the highest when the sodium alginate concentration was 2%. When the concentration of sodium alginate was too low, it resulted in low gel viscosity and failure to effectively form a stable structure. When the concentration of sodium alginate was too high, the strength of the CAMs increased, and their surface became denser [24], and thus *Microbacterium* sp. was not easily embedded. This study found that the deltamethrin removal rate of the CAMs was the highest when the CaCl_2_ concentration was 2%. When the concentration of Ca^2+^ is low, the gel structure cannot be compacted, and as a result, bacterial leakage can occur. When the Ca^2+^ concentration was higher than necessary, the pores of the gel surface became too small, which was not conducive to embedding bacteria [39]. This study found that the deltamethrin removal capability of the CAMs was the highest when the cross-linking time was 4 h. When the cross-linking time was too short, few bacteria entered the CAMs. When the cross-linking time was too long, the CAMs became denser, and the mass transfer resistance increased [24], thus preventing the entry of deltamethrin into the carrier for sufficient contact with *Microbacterium* sp.

Immobilized microbes have been used for the bioremediation of polluted sites with the advantages of easy separation, high mechanical strength, reusability, storability, and thermostability [16]. However, a few reports suggested that immobilization reduced the activity of microbes, and this has been attributed to the reduced intraparticle diffusion of contaminants [40,41]. In this study, the deltamethrin removal rate of *Microbacterium* sp.-containing CAMs was higher than that of free *Microbacterium* sp., whether in MS media, water, or soil, and the deltamethrin removal capability of *Microbacterium* sp.-containing CAMs was the highest in MS media, followed by that in water and soil. The observed low deltamethrin removal rate in water and soil may be explained by the necessity of bacterial adaptation to strange conditions [30]. The retardation in insecticide diffusion may also be related to the low deltamethrin removal rate in soil. Furthermore, the complex environment of soil, including the intense competition from indigenous microbes, phages’ phagocytosis, and the toxicity of toxic substances [42], all contribute to a lower deltamethrin removal rate of the CAMs. However, the water used in this study was simulated contaminated water, while real environmental contaminated water contains not only insecticide but also other pollutants. As some bacteria have been reported to be capable of degrading a wide spectrum of synthetic pyrethroids due to the high similarity in their chemical structure [30], future research should investigate the bacterial removal capability for other pollutants. Furthermore, the tested deltamethrin concentration in this study was relatively high, but it may be rather lower under complex natural conditions, and other carbon sources coexisting in the environment may competitively inhibit the degradation function of the target bacterial strains. Thus, it is necessary to systematically evaluate the insecticide removal efficacy of this strain in various contaminated environments.

This study found that immobilization with CAMs enhanced the deltamethrin removal capability of *Microbacterium* sp. and the possibility of recycling and reusing the bacteria many times. The immobilized bacteria had good reusability, and the deltamethrin removal efficiency still reached 50% after five reuse cycles. Thus, the reuse of *Microbacterium* sp.-containing CAMs reduces operating costs and increases economic benefits, showing high potential for the treatment of deltamethrin-contaminated water and soil. However, the research on immobilized microbial technology is still at the laboratory level; potential barriers and limitations preventing the widespread practical application of this technology, such as the shelf-life or long-term storage stability of the CAMs [24], should be gradually addressed in the future. As pyrethroids can produce more toxic metabolites from biodegradation [30], the chemical composition and the toxicity of deltamethrin derivates by microbial degradation should be evaluated in the future. Systematic risk assessment, covering risk identification and quantification, dose–response determination, and exposure assessment, is necessary prior to its practical application [43]. Furthermore, the effects of actual environmental characteristics (such as pH, salinity, phenol toxicity, antibiotics, heavy metals, and sulfides) on the physicochemical stability of carrier materials and the activity of immobilized biomass should be further evaluated [44].

## 5. Conclusions

The insect-derived strain, *Microbacterium* sp., was highly effective in degrading deltamethrin. This bacterial strain grew well in LB media within a large range of temperatures (20–40 °C) and pHs (5–9), and grew fastest in LB media with an inoculum volume of 3% in pH 7 at 175 rpm, 25 °C. Immobilization with sodium alginate enhanced its deltamethrin removal capability. *Microbacterium* sp.-containing CAMs generated with 2% sodium alginate and 3% CaCl_2_ cross-linking for 4 h exhibited the maximum deltamethrin removal capability and good reusability. Furthermore, *Microbacterium* sp.-containing CAMs showed a higher deltamethrin removal capability in deltamethrin-contaminated water and soil compared to free bacteria. These results suggest that *Microbacterium* sp. has great potential for the remediation of deltamethrin-contaminated environments. However, pesticide-contaminated environments in nature are significantly distinct from the aqueous and soil matrices utilized in this study. Subsequent research should therefore validate these findings under more field-relevant conditions that better represent actual environmental settings. Moreover, a systematic assessment of the biosafety of the bacterial strain’s degradation products, along with the potential risks associated with its large-scale environmental release, remains essential to confirming its ecological safety and technical feasibility.

## Figures and Tables

**Table 1:** Variables and levels for orthogonal test to optimize culture conditions of *Microbacterium* sp.

Variable	Level		
	1	2	3
Inoculum volume (%)	2	3	4
Temperature (°C)	20	25	30
Rotation speed (rpm)	125	150	175
pH	7	8	9

**Table 2:** Variables and levels for the orthogonal test to immobilize *Microbacterium* sp. with sodium alginate.

Variable	Level		
	1	2	3
Sodium alginate concentration (%)	1	2	3
CaCl_2_ concentration (%)	2	3	4
Cross-linking time (h)	2	3	4

**Table 3:** Extreme difference analysis of OD_600_ values of the bacterial solution from the orthogonal test to optimize culture conditions of *Microbacterium* sp.

No.	Inoculum Volume	Temperature	Rotation Speed	pH	OD_600_
1	1	1	1	1	1.738
2	1	2	2	2	1.850
3	1	3	3	3	1.597
4	2	1	2	3	2.063
5	2	2	3	1	2.283
6	2	3	1	2	1.692
7	3	1	3	2	2.060
8	3	2	1	3	1.742
9	3	3	2	1	1.843
K1	5.185	5.861	5.172	5.864	
K2	6.038	5.875	5.756	5.602	
K3	5.645	5.131	5.939	5.402	
k1	1.728	1.954	1.724	1.955	
k2	2.013	1.958	1.919	1.867	
k3	1.882	1.710	1.980	1.801	
R	0.285	0.248	0.256	0.154	

**Table 4:** Extreme difference analysis of deltamethrin removal rates from the orthogonal test to optimize the conditions to immobilize *Microbacterium* sp. with sodium alginate.

No.	Sodium Alginate Concentration	CaCl_2_ Concentration	Cross-Linking Time	Blank	Deltamethrin Removal Rate (%)
1	1	1	1	1	56.4
2	1	2	2	2	57.1
3	1	3	3	3	55.8
4	2	1	2	3	72.3
5	2	2	3	1	76.4
6	2	3	1	2	71.3
7	3	1	3	2	65.6
8	3	2	1	3	66.2
9	3	3	2	1	62.4
K1	169.3	194.3	193.9	195.2	
K2	220.0	199.7	191.8	194.0	
K3	194.2	189.5	197.8	194.3	
k1	56.4	64.8	64.6	65.1	
k2	73.3	66.6	63.9	64.7	
k3	64.7	63.2	65.9	64.8	
R	16.9	3.4	2.0	0.4	

## Data Availability

Data will be made available on request.
